# Dissolution–Precipitation Using Natural Terpenes as Pretreatment for PET Enzymatic Depolymerization

**DOI:** 10.1021/acsomega.5c13648

**Published:** 2026-06-13

**Authors:** João V. M. Resende, Sofia C. Aparício, Isabel M. Marrucho, Bernardo Dias Ribeiro

**Affiliations:** † CleanTech − Clean Technology Laboratory, School of Chemistry, Federal University of Rio de Janeiro, Avenida Athos da Silveira Ramos, 21941-598 Rio de Janeiro, Brazil; ‡ Centro de Química Estrutural and Institute of Molecular Science and Departamento de Engenharia Química, Instituto Superior Técnico, Universidade de Lisboa, Avenida Rovisco Pais, 1049-001 Lisboa, Portugal

## Abstract

Plastics are widely used in short-lived applications but pose a significant environmental challenge, emphasizing the need for efficient recycling strategies. Poly­(ethylene terephthalate) (PET) remains a major focus of these efforts, yet its high crystallinity continues to hinder recycling efficiency across mechanical, chemical, and enzymatic routes. Enzymatic depolymerization offers a sustainable pathway for monomer recovery due to its mild operating conditions and simplified downstream processing. This study reports a green pretreatment strategy for PET using a thymol:carvacrol (1:1) mixture through dissolution–precipitation. A full factorial design was employed to evaluate the influence of dissolution temperature, polymer concentration, and solvent removal rate on crystallinity reduction. Although none of the factors were individually significant within the tested ranges, all treatments reduced PET crystallinity by up to 14.1% without altering the chemical structure of the polymer. Pretreated samples exhibited markedly enhanced enzymatic depolymerization, producing approximately seven times more monomers than untreated PET after 72 h using HiC. These results demonstrate that this natural solvent-based pretreatment effectively lowers crystallinity and substantially improves enzymatic hydrolysis. The approach provides a promising route to enable more efficient PET upcycling and supports the development of greener, more sustainable recycling technologies.

## Graphical abstract

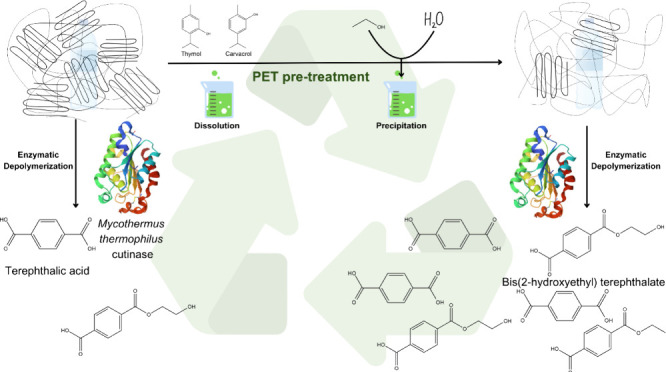

## 1 Introduction

Plastics are versatile, low-cost materials with widespread applications, resulting in an annual global production exceeding 360 million tonnes.^1^ However, owing to their chemical stability and long environmental persistence, mismanaged plastics do not receive adequate postconsumer treatment and thus accumulate in ecosystems.^2^ Among these materials, poly­(ethylene terephthalate) (PET) stands out as one of the most widely used plastics, particularly in textiles, beverage bottles, and packaging. PET also exhibits comparatively high recycling rates, especially in Brazil^3^ and Europe,^1^ where more than 40% of postconsumer PET is recycled annually.^4^ Although mechanical recycling remains the most straightforward and commonly applied strategy for PET waste management, it inevitably leads to downcycling. Repeated processing induces chain scission, lowering the polymer’s molecular weight^5,6^ and altering its physicochemical properties. This degradation reduces mechanical performance and ultimately compromises material circularity.^6^ Consequently, recycling strategies capable of recovering PET monomers or short oligomers are increasingly preferred, as they enable the production of new, pristine polymers or allow these molecular building blocks to be integrated into other value-added processes,^7,8^ offering a more sustainable pathway for PET waste valorization.

Chemical recycling technologies are promising, but they often rely on expensive or synthetically complex catalysts^9^ and require high operating temperatures (typically 100–250 °C),^10–12^ conditions that run counter to the principles of green chemistry. To overcome these limitations, while still enabling monomer recovery, recent research has increasingly turned toward the enzymatic depolymerization of PET.^13–17^ This strategy retains the advantages of chemical recycling, namely the ability to recover monomers, yet operates under much milder and more environmentally compatible conditions.^13^ A critical factor affecting the efficiency of enzymatic depolymerization, regardless of the enzyme employed, is PET crystallinity. Highly crystalline regions are structurally more rigid, limiting enzyme accessibility^18–20^ and contributing to a longer lag phase, ultimately decreasing hydrolysis rates as crystallinity increases.^21^ Consequently, reducing polymer crystallinity is a fundamental prerequisite for enhancing the overall efficiency of enzymatic PET depolymerization.

Several pretreatment methods have been proposed to achieve lower crystallinity, including micronization combined with extrusion or melt-extrusion, which can effectively reduce crystallinity in PET and other polyesters.^22–24^ However, like thermochemical recycling, these approaches rely on high temperatures. Other physical pretreatments, such as UV irradiation and ultrasound, may seem promising at first but have been shown to increase PET crystallinity, ultimately hindering enzymatic hydrolysis.^13^ Microwave-assisted pretreatment has emerged as a potential alternative, although it remains in its early stages and still requires further development and optimization.^25^ Polymer solubilization followed by controlled precipitation represents a promising strategy to facilitate PET depolymerization, as the process can significantly alter the polymer’s physical structure, making it more accessible to chemical or enzymatic attack.^26^ When PET is dissolved, its crystalline and amorphous regions are disrupted at the molecular level. Upon precipitation, PET typically resolidifies with lower crystallinity, reduced long-range order, and increased surface heterogeneity.^27^ Moreover, dissolution–precipitation can aid in removing additives, dyes, and other contaminants present in postconsumer plastics,^28^ generating a cleaner and more reactive substrate. Thus, this approach modifies PET morphology, while simultaneously improving enzyme–substrate interactions and accelerating depolymerization kinetics.

Among the solvents proposed in the literature for dissolution of polymers, eutectic solvents formulated from renewable, biodegradable components, as well as other mixtures of natural compounds, are considered greener alternatives to conventional petroleum-derived solvents. Due to their intrinsic mixture characteristics, these solvents possess highly tunable physicochemical properties and can be tailored as task-specific solvents for targeted applications.^29,30^ In addition, several of eutectic solvents and other mixtures of natural compounds have demonstrated compatibility with enzymatic and microbial systems, where they may activate or stabilize enzymes and enhance catalytic performance, thereby broadening their utility in biocatalytic and bioprocessing settings.^31,32^ These capabilities stem from the inherent physicochemical behavior of these natural mixtures, positioning them as versatile tools for developing more sustainable and effective PET pretreatment and depolymerization strategies.

Our previous work demonstrated that a 1:1 mixture of thymol and carvacrol possesses remarkable solubilization capacity for PET,^33^ even at lower temperatures than other natural or biobased solvents, such as γ-valerolactone^34^ and dimethyl isosorbide.^35^ Building on this finding, the present study uses this solvent mixture to develop a dissolution–precipitation pretreatment protocol to reduce PET crystallinity. To optimize the pretreatment conditions, the influence of three independent experimental factors, dissolution temperature, polymer concentration, and solvent-removal methodology, on the resulting polymer crystallinity was evaluated using a full factorial experimental design. The impact of the optimized pretreatment on PET depolymerization was subsequently assessed using *Mycothermus thermophilus* cutinase (HiC),^36^ a commercially available enzyme that is among the most extensively investigated for PET hydrolysis.^14,15,32,36^ From a sustainability standpoint, enzymatic depolymerization must rely on biocatalysts that can be produced at scale with low environmental burden and stable performance across multiple operating cycles. Significant advances have been recently made in the enzymatic depolymerization of PET, including the discovery of new enzymes and the optimization of reaction conditions.^37^ Enzymes such as DuraPETase,^38^ TS-PETase,^39^ and FAST-PETase^15^ have demonstrated highly promising performance under optimized laboratory conditions. However, their current production constraints, such as high resource demand, limited manufacturing capacity, and reliance on specialized expression systems, pose challenges for their incorporation into large-scale, circular recycling infrastructures. Thus, these PETases have not yet reached bulk production or commercial availability. These limitations directly influence the life-cycle impacts and techno-economic viability of enzymatic PET recycling, given that enzyme availability and cost remain major contributors to process sustainability. In this context, the use of commercially available enzymes such as HiC provides a practical baseline for assessing pretreatment strategies and evaluating how solvent-based approaches may enhance depolymerization under conditions more representative of sustainable industrial implementation.

## 2 Experimental Section

### 2.1 Materials and Equipment

Regular water PET bottles were obtained in a local supermarket. Thymol (99%), bis­(2-hydroxyethyl) terephthalate (BHET, 98.7%), terephthalic acid (TPA, 98%), formic acid (>98%), and acetonitrile (99.9%) were purchased from Sigma-Aldrich (Germany), carvacrol (98%) from Tokyo Chemical Industries (Japan), ethanol (>99.8%) from Laborspirit (Portugal). Mono-(2-hydroxyethyl) terephthalate (MHET) was synthesized (>99% purity) by enzymatic hydrolysis as previously described.^36^ A *Mycothermus thermophilus* cutinase (HiC) enzyme solution was kindly donated by Novozymes (Araucária, Brazil).

### 2.2 Experimental Design

A full factorial design with 4 central points was developed, evaluating 2 quantitative continuous variables (dissolution temperature and polymer concentration) and a qualitative variable (solvent removal technology—slow or fast). These terms will be further explained in the PET Pretreatment subsection. A total of 12 experiments were conducted as summarized in Table 1, following the random disposal originated from the design of experiments. Runs marked as (C) refer to the central points.

**Table 1:** Full Factorial Experimental Design

Standard run	Temperature (°C)	Polymer concentration (%)^a^	Solvent removal methodology
TC5	130	5.0	Fast
TC7	130	10.0	Fast
TC12 (C)	140	7.5	Fast
TC10 (C)	140	7.5	Fast
TC6	150	5.0	Fast
TC8	150	10.0	Fast
TC1	130	5.0	Slow
TC3	130	10.0	Slow
TC11 (C)	140	7.5	Slow
TC9 (C)	140	7.5	Slow
TC2	150	5.0	Slow
TC4	150	10.0	Slow

a (mass_polymer_/mass_solvent_) × 100.

These ranges were chosen following our previous work,^33^ while also aiming to optimize operational conditions and enhance interactions between polymer and ES. While the dissolution temperature was previously reported only at 150 °C, in the present work temperatures between 130 and 150 °C were tested, since those temperatures allowed the dissolution time to remain in the same magnitude order, and a temperature reduction would be beneficial for the process as a whole regarding energy efficiency and greenness.

To analyze the significance of the crystallinity reduction observed in this experimental design, a t test (n = 3) was performed with a different PET bottle (%X_C_ = 37.44 ± 1.68), using the TC7 treatment as an example. This data is presented in Table S1.

### 2.3 Preparation of the Solvent Mixture

To prepare the solvent mixture for PET pretreatment, thymol and carvacrol were weighed and mixed at (1:1) molar proportion with continuous stirring at 60 °C for 15 min, until a clear liquid was obtained. Then, the mixture was allowed to cool down to room temperature slowly.^34^

### 2.4 PET Pretreatment

A PET bottle was cut into regular, round pieces with a paper hole puncher (6 mm). The polymer pieces were then solubilized following the conditions previously described. When the polymer pieces were completely dissolved, polymer precipitation was carried out through two distinct methodologies: fast and slow solvent removal.

For fast solvent removal, the polymer was precipitated using an ethanol and water solution (60% v/v) as antisolvent (AS), following the procedure described by Pestana et al.,^33^ at a ratio of 5 mL AS per gram of solvent. The mixture was then vortexed for 10 s and subsequently filtered under vacuum. The resulting polymer flakes were dried in an oven at 70 °C until a constant mass was reached. The dried polymer was then ground using liquid N_2_, washed with pure ethanol to remove residual solvent, and dried once more at 70 °C until constant mass was achieved.

In the slow solvent removal strategy, the PET solution was transferred to an Eppendorf tube, and precipitation was induced using the same ethanol and water solution 60% (v/v) as the antisolvent, in a proportion of 1 mL AS per gram of solvent. The mixture was centrifuged at 6,000 rpm for 5 min, after which the supernatant was discarded. The recovered PET was then washed with 96% ethanol in a Thermomixer (Eppendorf) at 70 °C for 10 min. This step aimed to gradually remove solvent entrapped within the polymer structure by increasing PET chain mobility at high temperature. The suspension was centrifuged again at 6,000 rpm, and this washing–centrifugation cycle was repeated a total of ten times for each sample. The recovered PET was subsequently dried in an oven at 40 °C until constant mass. Finally, the polymer was ground using liquid N_2_, washed with pure ethanol to complete solvent removal, and dried once more at 40 °C to constant mass.

While the fast method involves fewer steps and has previously been used by our group to treat PET,^33^ the slow method may induce different structural organization effects, such as changes in crystallinity, due to possible polymer chain cohesion.^40^ This may result from the longer residence time of the solvent within the polymer matrix during the slow procedure, allowing it to act as a plasticizer.^41^ Under these conditions, the solvent may disrupt the polymer structure, promoting amorphization,^42^ and can subsequently be removed, without interfering with the remaining steps of the process.

It should be noted that, although solvent and antisolvent recycling were not assessed in this study, we have previously demonstrated that complete recovery and reuse of both the solvent and the AS are feasible following PET dissolution when precipitation is carried out using the fast method.^33^

### 2.5 PET Characterization

#### 2.5.1 Fourier Transform Infrared (FTIR) Spectroscopy

FTIR spectra of the PET samples before and after solvent pretreatment were acquired using a PerkinElmer UATR Two ATR-FTIR spectrometer. Each spectrum was recorded with 8 scans over the range of 4000–400 cm^–1^ at a resolution of 4 cm^–1^.

#### 2.5.2 Thermogravimetric Analysis (TGA)

TGA was used to assess the thermal stability of PET and detect potential solvent contamination. Analyses were conducted using a HITACHI STA7200 thermal analysis system, in which approximately 5 mg of sample was heated to 600 °C at a rate of 10 °C min^–1^ under a nitrogen flow of 200 mL min^–1^.

#### 2.5.3 Differential Scanning Calorimetry (DSC)

DSC was used to determine PET crystallinity and melting temperature (T_m_) before and after the dissolution–precipitation pretreatment. Analyses were performed using a DSC 200 F3 (NETZSCH) calorimeter and consisted of two consecutive heating and cooling cycles. Samples were heated from 20 to 300 °C at a rate of 10 °C min^–1^, followed by cooling to 20 °C at 20 °C min^–1^, under a nitrogen flow of 50 mL min^–1^. Data from both cycles were used to determine crystallinity values.

PET crystallinity was calculated by comparing the melting enthalpy of 100% crystalline PET with that of the recovered PET using eq 1:^21^

1 $$X_{c}\left(\%\right)=\frac{\Delta H_{m}-\Delta H_{cc}}{\Delta H_{m}^{0}}\times 100$$

where *ΔH*_*m*_ is the sample’s melting enthalpy, *ΔH*_*cc*_ is the sample’s cold crystallization enthalpy, and *ΔH*_*m*_^0^ is the heat of melting of a pure PET crystalline sample, which is 136 J/g.^43^

#### 2.5.4 Gel Permeation Chromatography

The molecular weight of pristine PET and recovered samples was determined using an LF-804 column (Shodex) equipped with an LF-G precolumn (Shodex), both maintained at 40 °C. A mixture of HFIP and CHCl_3_ (2:98 v/v) was used as the mobile phase at a flow rate of 0.5 mL min^–1^. Detection was performed using a UV detector set at 254 nm in combination with an RI detector. The system was calibrated with 12 polystyrene standards ranging from 162 to 500,000 Da. Samples were prepared at 1 mg mL^–1^ by dissolving PET in HFIP and subsequently diluting with CHCl_3_ to obtain a final HFIP:CHCl_3_ ratio of 4:96 (v/v). Prior to analysis, all solutions were filtered through 0.45 μm PTFE filters (Merck Millipore).

### 2.6 PET Depolymerization Assays

The depolymerization reaction was carried out using HiC at an enzyme loading of 0.01 g_protein_/g_PET_ (128 U/mg_protein_). Reactions were performed with 4 mg of PET in a 10 mL reactor (1 g L^–1^ PET concentration) at 60 °C and 125 rpm for 72 h. The reaction medium consisted of 100 mM phosphate buffer supplemented with 160 μL of 5% sodium azide. The depolymerization products—BHET, MHET, and TPA—were quantified by high-performance liquid chromatography (HPLC) using a Shimadzu (Japan) system equipped with a Zorbax Eclipse Plus C18 column (250 × 4.6 mm, Agilent, USA). A gradient of acetonitrile and 0.05% formic acid served as the mobile phase at a flow rate of 0.5 mL min^–1^. The column temperature was maintained at 30 °C, and the injection volume was 10 μL. Detection of the hydrolysis products was carried out using a UV detector set at 254 nm with a cell temperature of 40 °C.

## 3 Results and Discussion

### 3.1 PET Pretreatment

The polymer dissolution time varied as a function of the temperature used in the experiments. All samples processed at 150 °C were fully solubilized after 7 min, as previously reported.^33^ Samples processed at 140 °C required 10 min for complete dissolution. At 130 °C, most samples were fully solubilized after 15 min. However, sample TC5 displayed a noticeably slower dissolution profile, with approximately 90% of the polymer dissolved at 15 min and the remaining 10% requiring a total of about 45 min to solubilize. Since PET pieces were randomly selected from the same bottle, the reason for this extended dissolution time is not clear, particularly given the many factors that influence polymer dissolution—such as molecular weight, structural organization, and chain conformation.^44^ It should be noted that all these dissolutions happen at a lower temperature and shorter time than the most significant parallels in literature, which are dissolutions in γ-valerolactone^26,34^ and isopropylphenol (180 °C)^26^ and dimethyl isosorbide (160 °C).^35^

Thermal characterization plays a central role in polymer science. Differential scanning calorimetry (DSC) is commonly used to evaluate changes in polymer chain properties, such as crystallinity, following processing or treatment, including curing operations.^45^ DSC is a reliable technique for crystallinity assessment, even when compared to the other widely used technique for this analysis, XRD.^46^ Thermogravimetric analysis (TGA), in turn, provides insights into polymer thermal stability and composition, since it can also confirm the absence of unwanted reaction compounds during thermal treatments used in recycling processes, thereby supporting the assessment of polymer quality.^47,48^ The thermal characterization parameters of the PET samples produced in this study by the two pretreatment methods are listed in Table 2. DSC was employed to calculate crystallinity (%X_C_) and to determine the melting (T_m_) and crystallization (T_c_) temperatures, while TGA was used to verify solvent removal efficiency and to evaluate the thermal decomposition behavior of the polymer, assessed here in terms of the main thermal decomposition temperature (T_d_).

**Table 2:** Thermal Characterization Parameters for the PET Samples Produced by the Two Pretreatment Methods Analyzed in This Study

	Dissolution temperature (°C)	Polymer concentration (%)^a^	Solvent weight loss (%)	T_d_ (°C)	T_c_ (°C)	T_m_ (°C)	ΔH_m_ (J g^–1^)	*X*_*c*_ (%)	Δ*X*_*c*_ (%)
	Untreated PET			418.3	197.4	246.5	64.1	47.1	–
Fast	130	5	0.48	411.9	196.5	246.1	59.3	43.6	–3.5
		10	0.31	420.7	196.3	245.3	54.6	40.2	–7.0
	140	7.5	0.31	418.3	195.7	246.0	50.9	37.4	–9.7
			0.31	418.3	194.1	248.3	48.3	35.5	–11.6
	150	5	0.31	418.7	195.5	246.7	51.2	37.6	–9.5
		10	0.31	418.5	196.9	249.6	52.1	38.3	–8.8
Slow	130	5	6.34	417.9	188.6	246.2	44.9	33.0	–14.1
		10	1.50	416.0	195.6	247.8	46.5	34.2	–12.9
	140	7.5	1.33	417.5	195.4	244.7	47.3	34.8	–12.3
			2.86	419.3	175.1	246.3	52.9	38.9	–8.3
	150	5	1.90	417.9	197.3	246.9	54.3	39.9	–7.2
		10	1.33	414.6	184.6	245.0	46.4	34.1	–13.0

a (mass_PET_/mass_solvent_) × 100.

Regardless of the treatment conditions applied, crystallinity decreased in all samples, with a maximum reduction of 14.1% observed for PET dissolved at 130 °C (TC1). The melting point remained essentially unchanged across all treated samples and relative to the pristine polymer, indicating little to no alteration in the polymer’s chemical structure (Figure S1). This interpretation is supported by the presence of a single decomposition peak in all TGA thermograms (Figure S2). It is also important to note that the PET samples crystallinity values obtained in this study are consistent with those reported in the literature.^49^

The crystallization temperature (T_c_) was generally slightly lower in the treated samples, except for TC1, TC4, and TC11, which exhibited markedly reduced T_c_ values compared with the remaining samples. This behavior may suggest that secondary crystallization, enabled by the thermal treatment applied after precipitation, occurred to a greater extent in these particular samples. Even so, their overall crystallinity remained lower than that of the original PET. Finally, no correlation was observed between T_c_ (or T_m_) and the crystallinity values.

An ANOVA test was performed to ascertain which are the determinant factors in PET amorphization and the results are presented in Table S2. Interestingly, none of the evaluated factors were statistically significant within the tested ranges, as indicated by the ANOVA results. It is also worth noting that some variation was observed among the central-point replicates, particularly for the slow solvent-removal methodology. This small variability can be in part caused by certain inherent characteristics of dissolution–precipitation processes. Small variations in precipitation kinetics, solvent retention, and local polymer heterogeneity can significantly influence how polymer chains reorganize during precipitation. Since nucleation and recrystallization are highly sensitive to subtle changes in mixing and temperature, even nominally identical conditions may yield polymers with slightly different morphologies or crystallinity levels. As a result, repeated dissolution–precipitation cycles tend to produce measurable variability in the final material structure.

Although the differences between treatment conditions were not statistically significant, the slow methodology generally produced greater crystallinity reductions (7–14%) compared with the fast method (3–12%). Regarding TGA data, no significant differences in thermal degradation behavior were observed across samples or relative to pristine PET. All samples exhibited a main decomposition peak around 417 ± 3 °C, except for TC5, which presented a T_d_ of 411.90 °C. Some samples present an initial mass loss below 150 °C, corresponding to residual solvent within the polymer samples. Collectively, these results indicate that no depolymerization occurred during the solvent pretreatment, as no additional decomposition peaks associated with monomers or oligomers were detected, as seen in Table S3.

It should be noted that the residual solvent content in the final polymer is higher for samples treated with the slow method than for those treated with the fast method (Table 2). As previously discussed, the solvent becomes more effectively trapped within the polymer matrix during the slow procedure due to the high number of dissolution–precipitation cycles. The initial hypothesis was that the solvent might occupy interstitial spaces within the polymer macrostructure and that, upon its slow, meticulous removal, these regions would remain unfilled, thereby increasing the overall amorphous content of the material. However, because complete solvent removal was not achieved, this hypothesis could not be fully evaluated. Nonetheless, it is important to emphasize that the presence of residual solvent does not correlate with crystallinity reduction, as it can be concluded from the analysis of Tables 1 and 2. Additionally, the presence of residual solvent does not appear to influence any of the other polymer properties assessed in this study.

FTIR analysis was performed to assess potential changes in the chemical structure of the recovered PET by comparing its spectrum with that of pristine PET (Figure 1). Since all the relevant and more intense peaks are observed in the 2000–400 cm^–1^ range, that is the range shown here. Still, the full spectra are available in the Supporting Information (Figure S3a–c).

**Figure 1:**
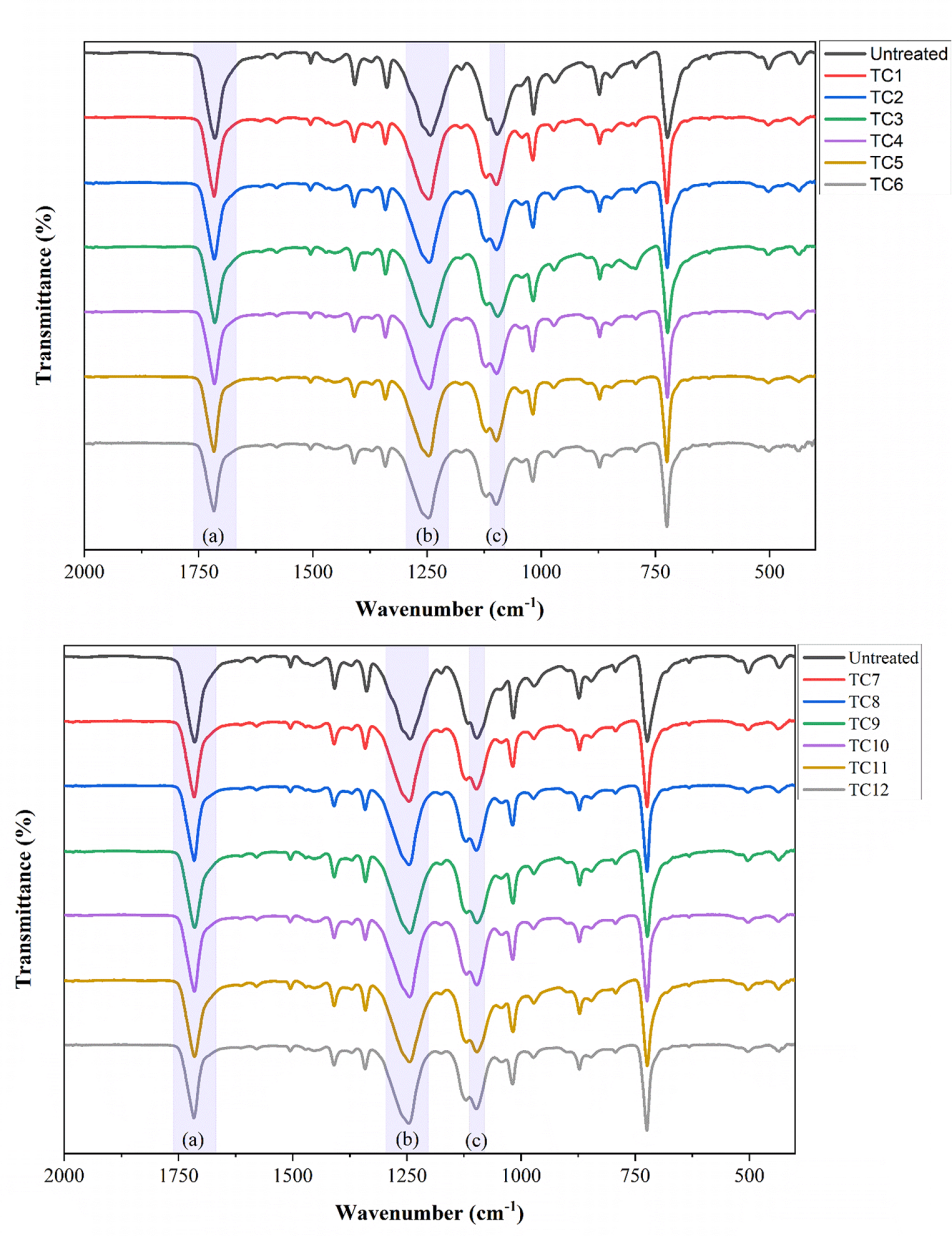
Magnification of the FTIR spectra in the 2000–400 cm^–1^ region for the untreated PET bottle and samples TC1–TC6 (top) and TC7–TC12 (bottom).

All samples, treated and untreated, exhibited the characteristic PET absorption bands, including 1714 cm^–1^ (ν­(C=O)), 1250 cm^–1^ (ν­(C–C–O–Ar)), and 1098 cm^–1^ (ν­(C–O–C)).^50^ No new peaks or peak disappearances were observed in the FTIR spectra, indicating that the polymer did not undergo chemical or structural changes at the atomic level during the solvent pretreatment and was affected only physically.

The molecular weight of representative PET samples was also evaluated before and after pretreatment. The pristine PET bottle exhibited an average molecular weight of 70 kg mol^–1^, while samples subjected to the fast and slow methodologies showed molecular weights of 72 and 68 kg mol^–1^, respectively. These differences correspond to a small variation of approximately 3%, and the molecular weight distributions were consistent across samples, as shown in Figure S4. Thus, the pretreatment does not induce significant depolymerization.

### 3.2 PET Enzymatic Depolymerization

Having established the effects of the solvent pretreatment on PET structure and morphology, the next step was to evaluate how these changes influence enzymatic depolymerization. To assess whether the observed crystallinity reduction was sufficient to enhance enzymatic depolymerization, an untreated PET sample and a thymol + carvacrol (1:1)-pretreated sample were subjected to hydrolysis by HiC, as a proof of concept. Among the three samples showing the highest crystallinity reduction (TC1, TC3, and TC4), TC3 was selected for enzymatic evaluation because it was processed at the lowest dissolution temperature (relative to TC4) and employed a higher polymer-to-solvent ratio (relative to TC1).

Figure 2 presents the concentrations of the two main depolymerization products, TPA and MHET, for both untreated and thymol + carvacrol (1:1)-pretreated PET. The pretreated sample exhibited a markedly enhanced depolymerization profile, producing approximately seven times more total products after 72 h (TPA: 27.69 mg L^–1^; MHET: 17.17 mg L^–1^) compared with the untreated polymer (TPA: 3.95 mg L^–1^; MHET: 4.40 mg L^–1^).

**Figure 2:**
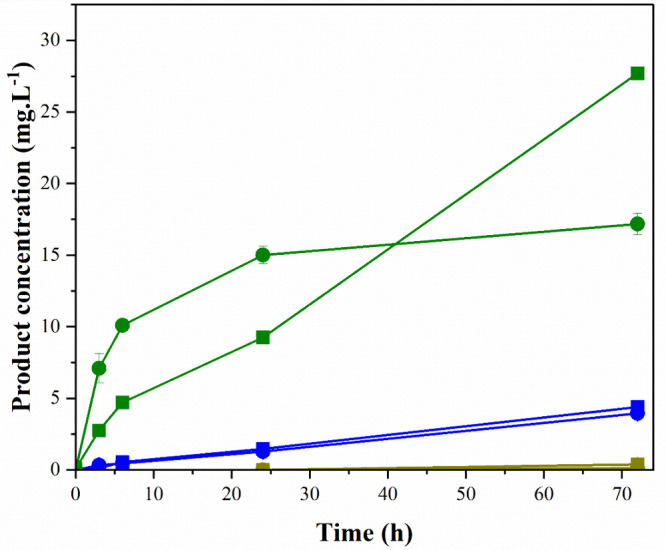
Evolution of enzymatic depolymerization product (TPA, MHET) concentration (mg L^–1^) with time. Squares: MHET; circles: TPA. Green: pretreated PET; blue: untreated PET; gold: blank.

It is noteworthy that the expected MHET molar fraction for this reaction after 72 h is 0.56,^35^ which was indeed observed for the untreated PET. In contrast, the pretreated sample displayed a lower MHET molar fraction of 0.38. This shift suggests that amorphization not only accelerates the overall depolymerization rate but may also alter the reaction kinetics to favor increased TPA production. Such an effect could be advantageous from an industrial perspective and merits further investigation.

As expected, BHET was not detected at any reaction time, as HiC rapidly converts BHET into MHET and ethylene glycol.^36^ No depolymerization products were observed in any sample at t = 0 h. A very small amount of hydrolysis products was detected in the blank sample at t = 72 h, which is consistent with the TGA and DSC results indicating that no depolymerization occurred prior to enzymatic treatment. The presence of oligomers can be inferred from additional peaks appearing at high retention times in the chromatograms. Although these peaks could not be identified or quantified due to the absence of analytical standards, they indicate that depolymerization was still progressing after 72 h. Such oligomers correspond to intermediate hydrolysis products that can be further cleaved to produce monomers.^52^ Their persistence also suggests that the reaction had not reached equilibrium within the 72-h period, which is consistent with the inherently slow kinetics reported for PET enzymatic depolymerization.^14^ It should be noted that the intent of this study is to shed light on the possibility of using a dissolution–precipitation treatment to reduce crystallinity, envisioning enzymatic depolymerization. Thus, the present enzymatic depolymerization section is not the main focus of the paper but rather a proof-of-concept demonstrating the application of this process to a real situation. Therefore, instead of the traditional 7+ days needed to reach equilibrium, allowing full evaluation of the reaction kinetics,^14,36,53^ this paper focuses only on the initial 3 days of the reaction, since those are more than enough to observe the impact of the pretreatment in the reaction kinetics, a major factor for industrial feasibility.

At this point, two different effects, polymer particle size and crystallinity, should be taken into consideration in the discussion of the factor that influence enzymatic depolymerization. Particle size mainly impacts the initial reaction rate, as smaller particles provide greater surface area for enzyme adsorption.^51^ Crystallinity, however, governs the long-term hydrolysis efficiency, since amorphous regions are readily accessible to enzymes while crystalline domains hydrolyze much more slowly.^18^ Thus, particle size influences how quickly the reaction starts, whereas crystallinity determines how far and how efficiently depolymerization can proceed. Reaction kinetics also help explain the substantial differences observed between the depolymerization of untreated and treated PET. Although crystallinity does not strongly influence enzymatic adsorption, it plays a pivotal role in the catalytic turnover rate, with more crystalline materials exhibiting slower reaction kinetics than amorphous ones.^14^ Particle size is another key factor affecting the reaction kinetics^14,51,54^ and, since this study does not reach the reaction equilibrium, it could contribute to differences in overall yields, as the untreated PET had slightly larger particles than the pretreated material. However, the magnitude of the increase in monomer production (upward to seven times) cannot be attributed to particle size alone. Rather, the combined effects of reduced crystallinity and smaller particle size act synergistically to accelerate the reaction kinetics, with the first one being the main driver.

Overall, the substantial depolymerization observed in this study is attributable to the enzymatic activity of HiC rather than to the reaction conditions. These results demonstrate that PET amorphization was a key factor enabling enhanced enzymatic depolymerization, by accelerating reaction kinetics as well as allowing for a greater depolymerization grade in general. The increase in product formation arises from HiC’s ability to directly attack the modified polymer structure, a behavior distinct from that reported in earlier studies.^25^

## 4 Conclusions

A mixture of natural occurring compounds, thymol:carvacrol (1:1), proved effective as a mild pretreatment for PET solubilization through a dissolution–precipitation approach, enabling polymer amorphization without altering its chemical structure or compromising its physicochemical integrity. The pretreatment consistently reduced PET crystallinity, a desirable outcome for enzymatic depolymerization processes. When subjected to hydrolysis by HiC, pretreated PET exhibited up to a 7-fold increase in monomer production compared with untreated PET, demonstrating that reduced crystallinity directly enhances enzymatic depolymerization efficiency.

The influence of treatment temperature, polymer concentration, and solvent removal methodology on the amorphization process was evaluated using a factorial experimental design. None of these parameters showed significant individual effects within the tested ranges, suggesting that additional, unstudied factors may govern crystallinity reduction during dissolution–precipitation. Even so, the results confirm that this pretreatment can reliably decrease PET crystallinity without inducing depolymerization.

Overall, the dissolution–precipitation strategy using this green solvent mixture represents a promising pretreatment to improve PET enzymatic depolymerization yields. Its integration into future PET recycling workflows may contribute to more efficient upcycling routes and support ongoing efforts to reduce plastic waste.

## Supplementary Material



## Notes

Full statistical data; extra TGA data for PET, BHET and oligomers decomposition from literature; full FTIR spectra (4000–400 cm^–1^) for all samples; and combined thermograms and molecular weight distribution of PET by gel permeation chromatography (PDF)
