# Supplementary material for: Dissolution–Precipitation Using Natural Terpenes as Pretreatment for PET Enzymatic Depolymerization
Source: ACS Omega. 2026 Jun 13;11(25):36792–800. doi: 10.1021/acsomega.5c13648 (PMC13325152; doi:10.1021/acsomega.5c13648)
Supplement: Supplementary file 1 [file ao5c13648_si_001.pdf]

Dissolution-Precipitation using natural terpenes as pre-treatment for PET enzymatic depolymerization

João V. M. Resende[a,b]*, *Sofia C. Aparício*[b], *Isabel M. Marrucho*[b], *Bernardo D. Ribeiro*[a]

[a] CleanTech – Clean Technology Laboratory, School of Chemistry, Federal University of Rio de Janeiro, Avenida Athos da Silveira Ramos, 21941-598 Rio de Janeiro, Brazil.

[b] Centro de Química Estrutural and Institute of Molecular Science and Departamento de Engenharia Química, Instituto Superior Técnico, Universidade de Lisboa, Avenida Rovisco Pais, 1049-001 Lisboa, Portugal.

* jvmresende@ufrj.br

SUPPORTING INFORMATION

Table S1. T-student test showing crystallinity difference between treated and untreated PET. df= 4, t=4.75, p=0.009. (DOC).

**Source**	**Crystallinity**
Pre-treatment PET	37.44 ± 1.68
Post-treatment PET	28.35 ± 2.49

Table S2. ANOVA test results for sample crystallinity percentile (DOC).

Source	Sum of squares	DF	Mean square	F value	Prob > F
(1)Temperature	0.1405	1	0.1404	0.02807	0.8824
(2)Concentration	6.7712	1	6.7712	1.35302	0.3648
(3) Solvent removal methodology (slow/fast)	25.9896	1	25.9896	5.19325	0.1503
1 by 2	1.0368	1	1.0368	0.20717	0.6936
1 by 3	26.5721	1	26.5720	5.30963	0.1477
2 by 3	0.4608	1	0.4608	0.09208	0.7902
1*2*3	15.4568	1	15.4568	3.08858	0.2209
Lack of Fit	19.1410	2	9.5705	1.91238	0.3434
Pure Error	10.0090	2	5.0045		
Total SS	105.5777	11			

Table S3. Decomposition temperature (T_d) of different products in PET TGA curve. Tests were carried out at 10°C min^{-1} heating rate (DOC).

**Product**	**T_d (°C)**	**References**	**Decomposition range (°C)**	**References**
PET	432-440	[1–5]	391-431;	[6]
			392-489	[7]
BHET	283.7	[5]	236.6 – 284.4	[6]
			244-355; 388	[7]
			210-270; 390-430 and 480-540	[8]
Oligomers	264.5	[5]	In-between PET and BHET	[8]

(1) Dimitrov, N.; Kratofil Krehula, L.; Ptiček Siročić, A.; Hrnjak-Murgić, Z. Analysis of Recycled PET Bottles Products by Pyrolysis-Gas Chromatography. *Polym. Degrad. Stab.* **2013**, *98* (5), 972–979. https://doi.org/10.1016/j.polymdegradstab.2013.02.013.

(2) Wojtyła, S.; Klama, P.; Baran, T. Is 3D Printing Safe? Analysis of the Thermal Treatment of Thermoplastics: ABS, PLA, PET, and Nylon. *J. Occup. Environ. Hyg.* **2017**, *14* (6), D80–D85. https://doi.org/10.1080/15459624.2017.1285489.

(3) Das, P.; Tiwari, P. Thermal Degradation Study of Waste Polyethylene Terephthalate (PET) under Inert and Oxidative Environments. *Thermochim. Acta* **2019**, *679*, 178340. https://doi.org/10.1016/j.tca.2019.178340.

(4) Tao, Y.; Liu, C.; Li, P.; Wang, B.; Xu, Y.-J.; Jiang, Z.-M.; Liu, Y.; Zhu, P. A Flame-Retardant PET Fabric Coating: Flammability, Anti-Dripping Properties, and Flame-Retardant Mechanism. *Prog. Org. Coat.* **2021**, *150*, 105971. https://doi.org/10.1016/j.porgcoat.2020.105971.

(5) Fang, P.; Lu, X.; Zhou, Q.; Yan, D.; Xin, J.; Xu, J.; Shi, C.; Zhou, Y.; Xia, S. Controlled Alcoholysis of PET to Obtain Oligomers for the Preparation of PET-PLA Copolymer. *Chem. Eng. J.* **2023**, *451*, 138988. https://doi.org/10.1016/j.cej.2022.138988.

(6) Pham, D. D.; Cao, A. N. T.; Senthil Kumar, P.; Nguyen, T. B.; Tran Nguyen, H.; Phuong, P. T. T.; Nguyen, D. L. T.; Nabgan, W.; Trinh, T. H.; Vo, D.-V. N.; Nguyen, T. M. Insight the Influence of the Catalyst Basicity on Glycolysis Behavior of Polyethylene Terephthalate (PET). *Chem. Eng. Sci.* **2023**, *282*, 119356. https://doi.org/10.1016/j.ces.2023.119356.

(7) Hu, Y.; Wang, Y.; Zhang, X.; Qian, J.; Xing, X.; Wang, X. Synthesis of Poly(Ethylene Terephthalate) Based on Glycolysis of Waste PET Fiber. *J. Macromol. Sci. Part A* **2020**, *57* (6), 430–438. https://doi.org/10.1080/10601325.2019.1709498.

(8) Enayati, M.; Mohammadi, S.; Bouldo, M. G. Sustainable PET Waste Recycling: Labels from PET Water Bottles Used as a Catalyst for the Chemical Recycling of the Same Bottles. *ACS Sustain. Chem. Eng.* **2023**, *11* (46), 16618–16626. https://doi.org/10.1021/acssuschemeng.3c04997.

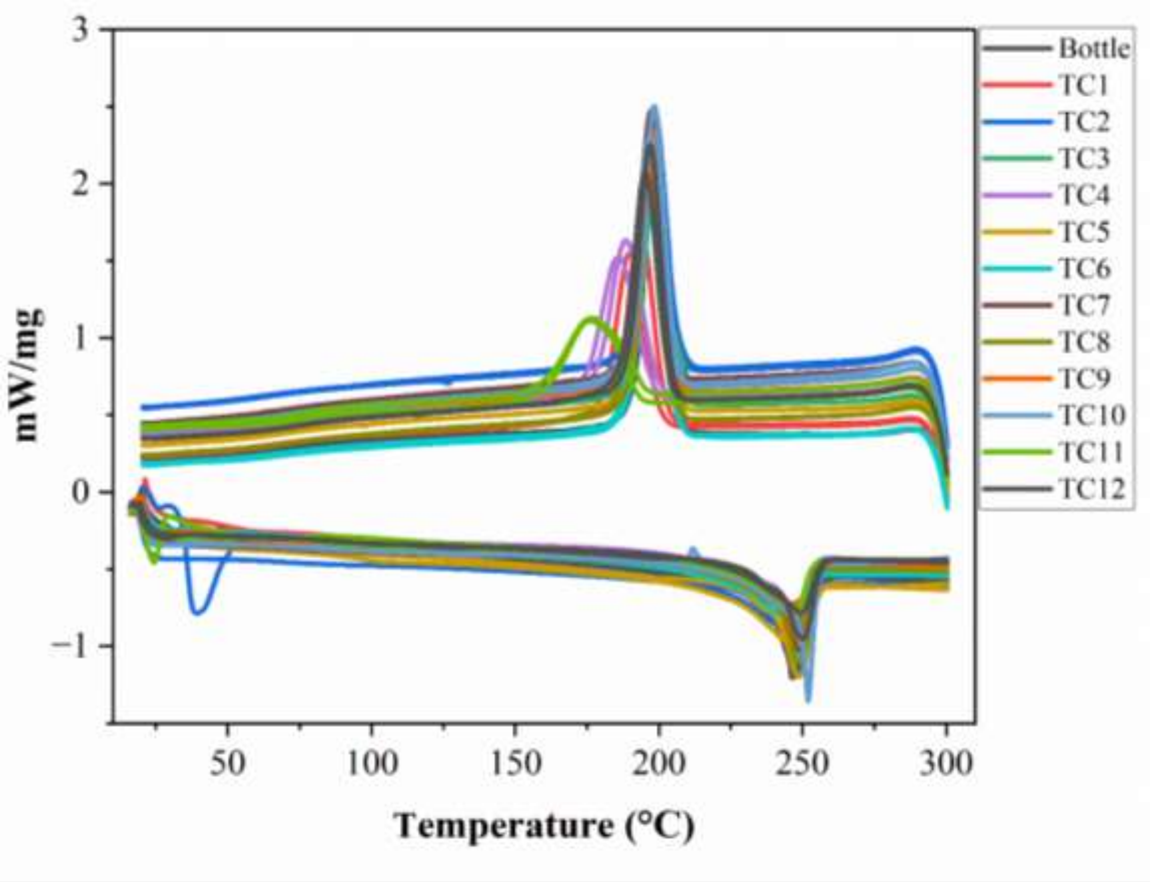

Figure S1: Combined DSC data of the polymer samples in this study (TIF).

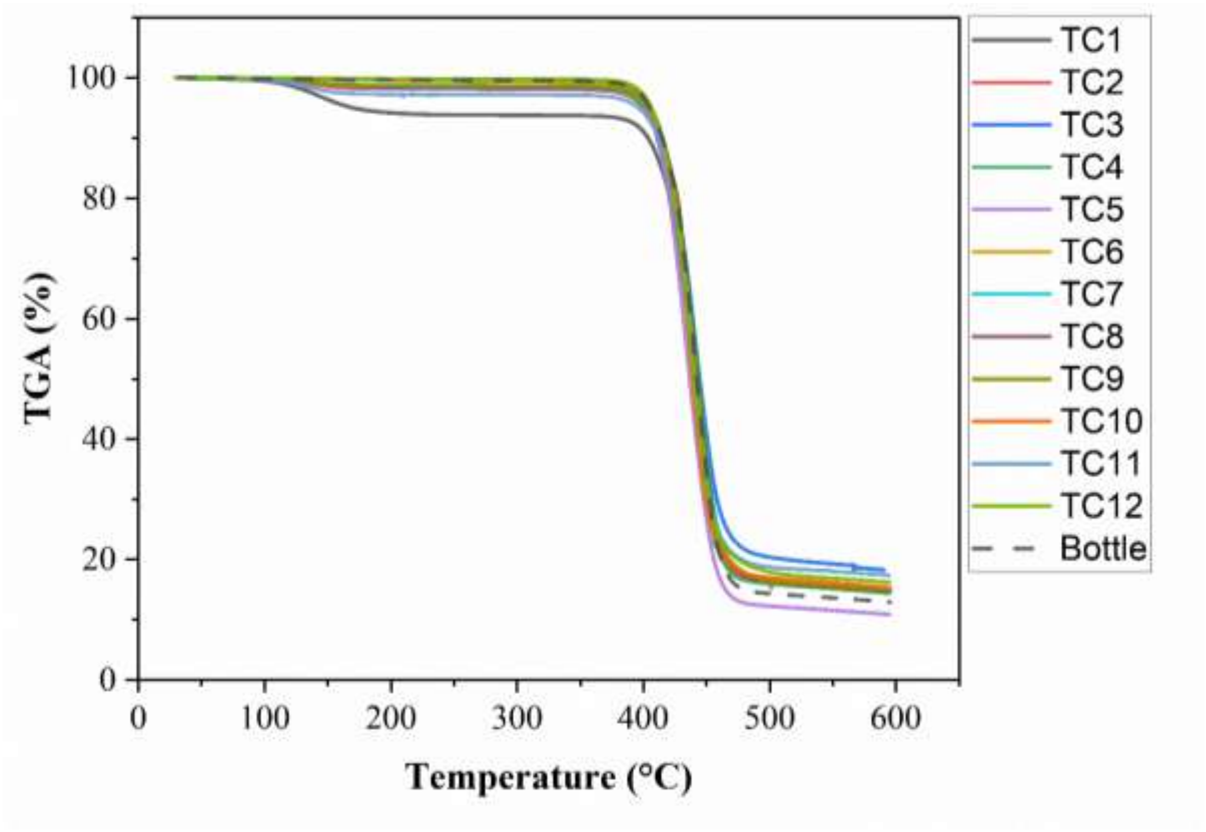

Figure S2: Combined thermogram of the polymer samples in this study (TIF).

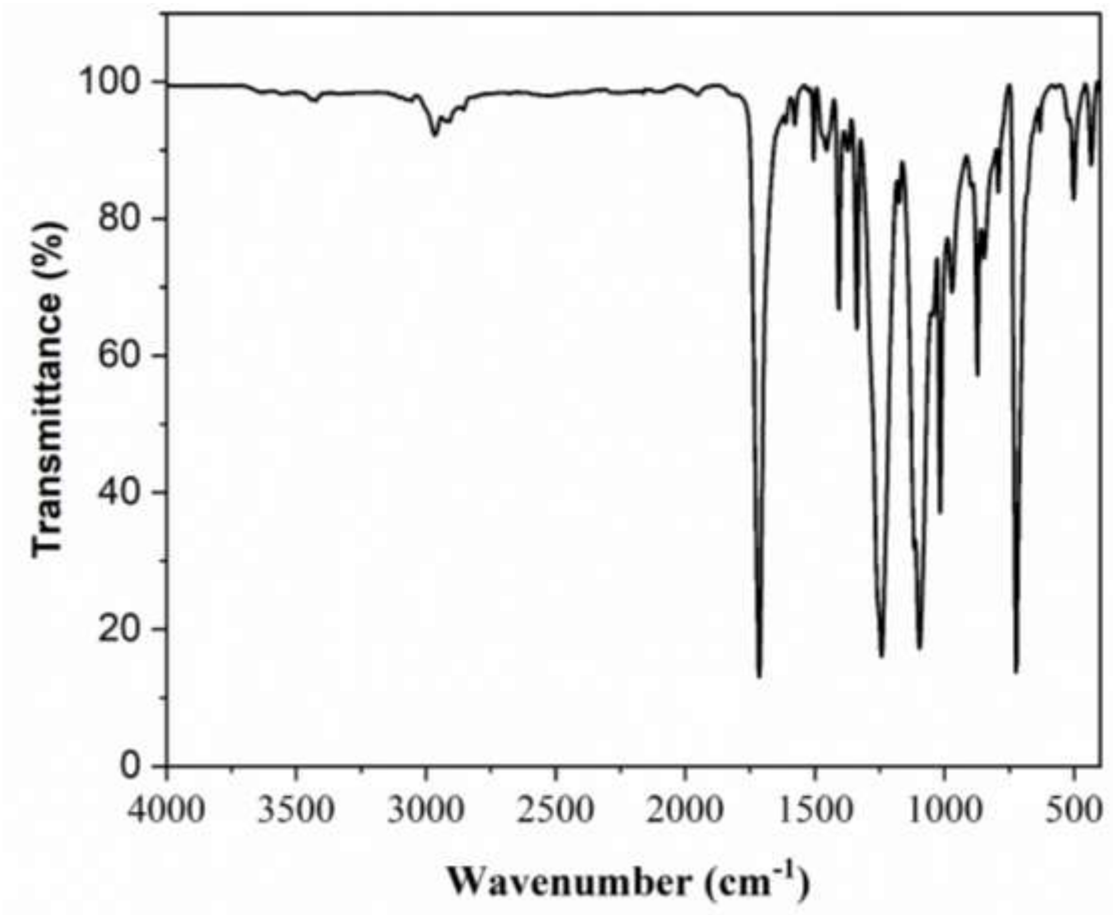

Transmittance (%)
100
80
60
40
20
0
4000
3500
3000
2500
2000
1500
1000
500
Wavenumber (cm-1)

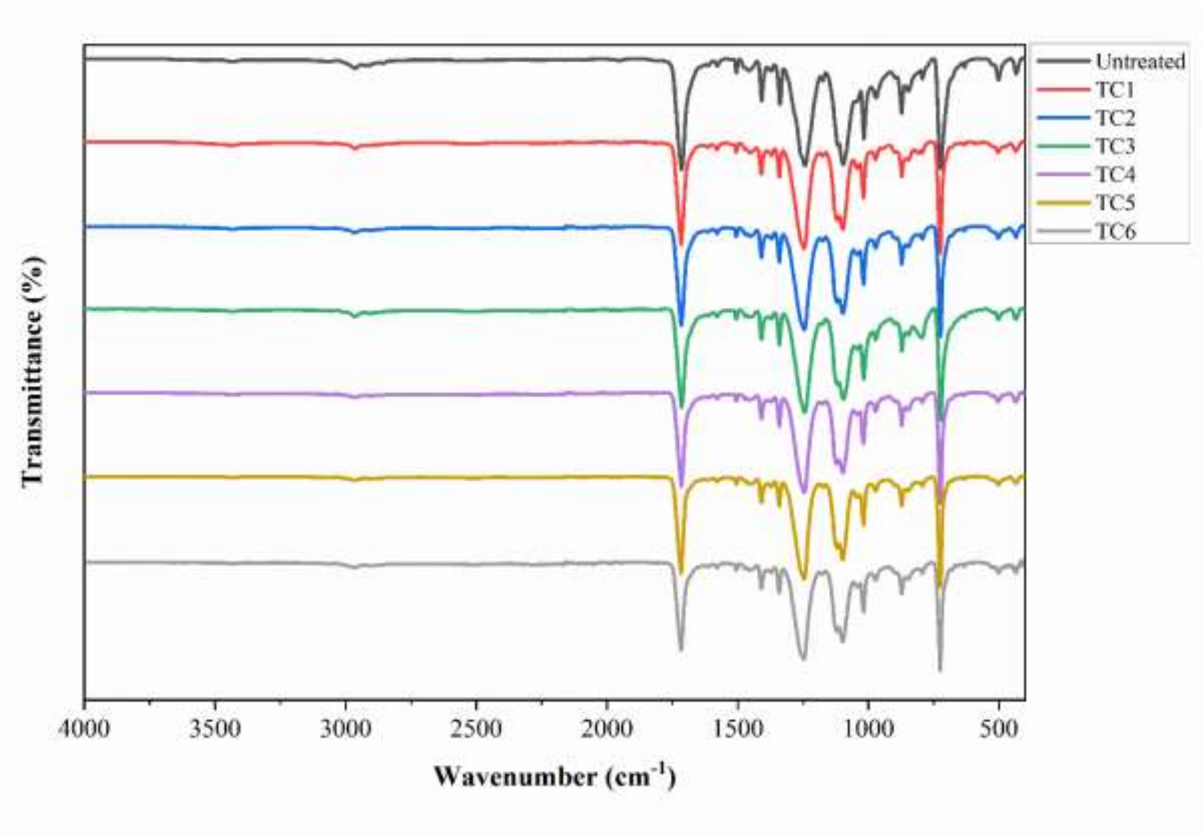

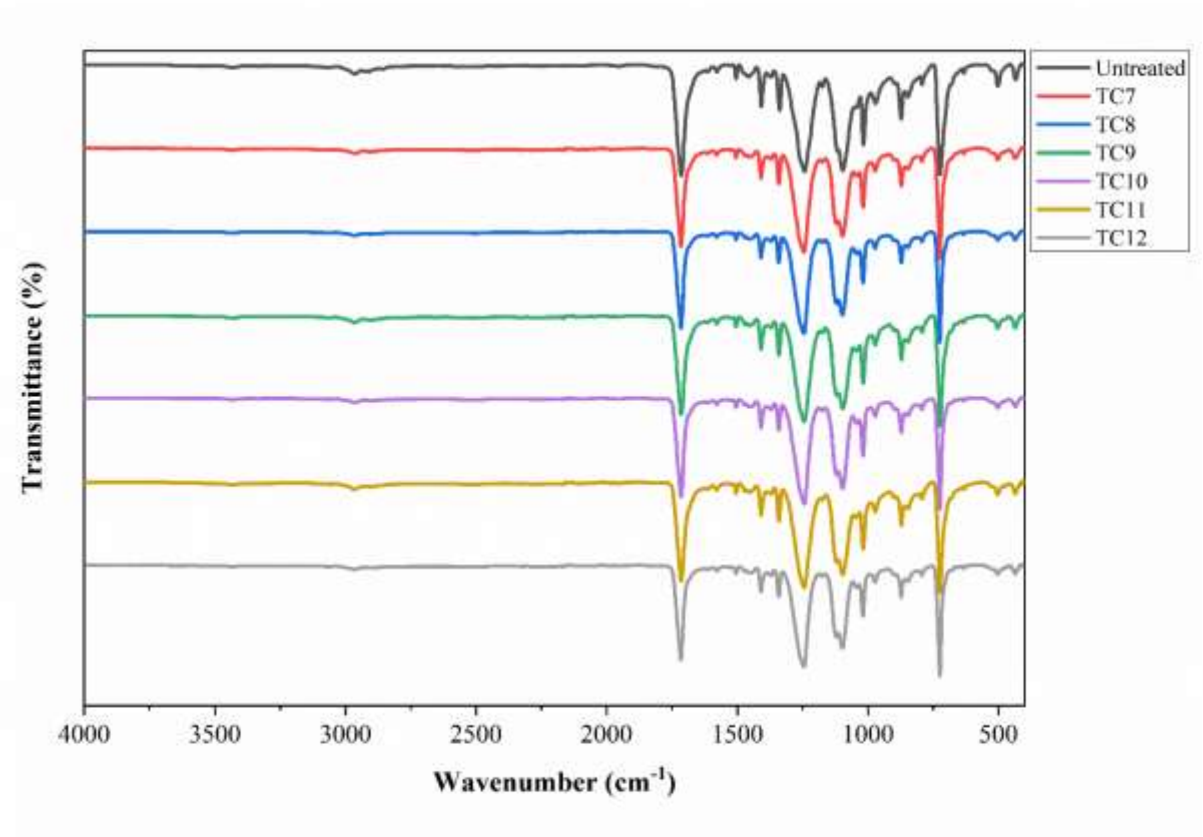

Figure S3: FTIR spectra of the untreated PET bottle (a) and of all the samples involved in this study (b, c). (TIF).

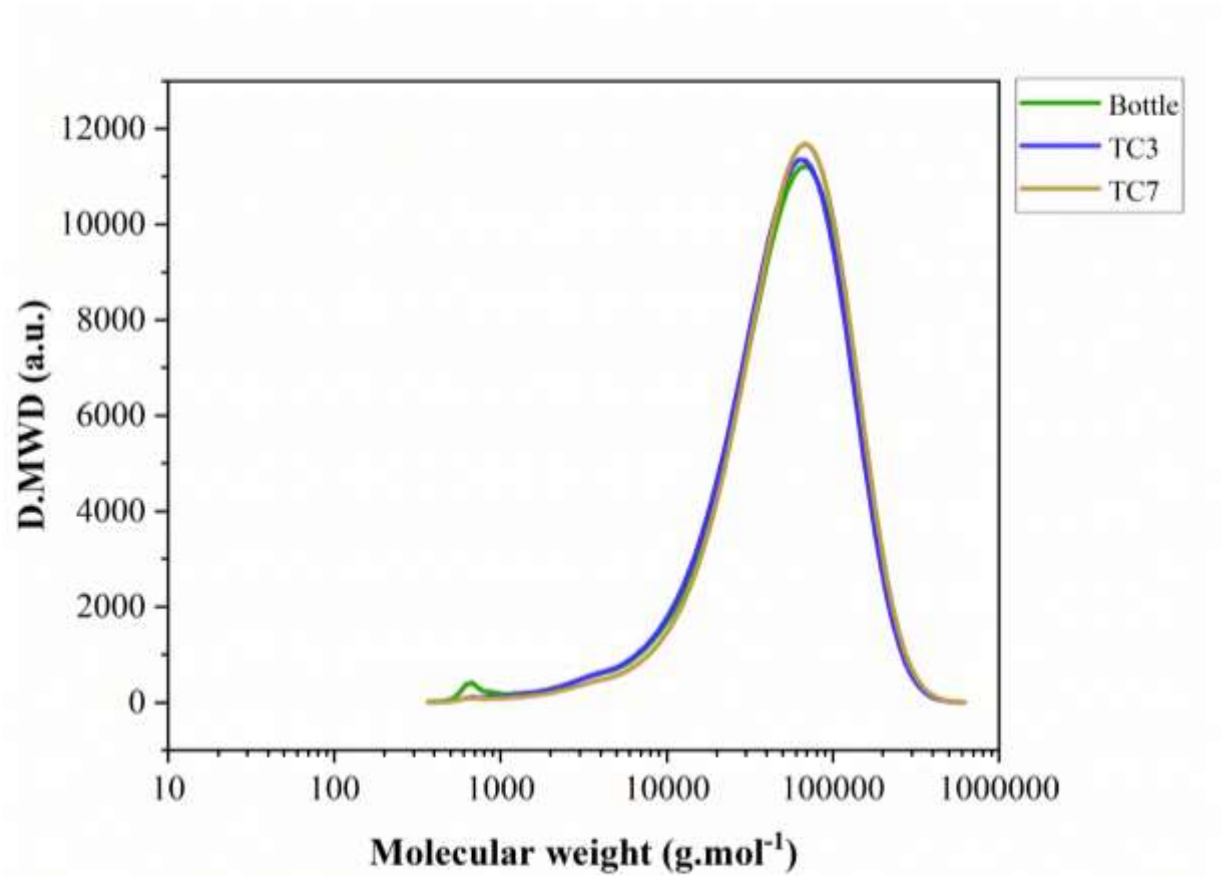

Figure S4: Molecular weight distribution of pristine and pretreated PET samples (TIF).
